# Decoding of Repeated Objects from Local Field Potentials in Macaque Inferior Temporal Cortex

**DOI:** 10.1371/journal.pone.0074665

**Published:** 2013-09-05

**Authors:** Dzmitry A.e Kaliukhovich, Rufin Vogels

**Affiliations:** Laboratorium voor Neuro- en Psychofysiologie, KU Leuven Medical School, Leuven, Belgium; National Institute of Mental Health, United States of America

## Abstract

Stimulus repetition produces a decrease of the response and affects neuronal synchronization of macaque inferior temporal (IT) neurons. Previously we showed that such stimulus-specific adaptation results in a decreased accuracy by which IT neurons encode repeated compared to non-repeated objects. Not only spiking activity, but also local field potentials (LFPs) are affected by repetition. Here we ask how the repetition-induced changes in IT LFPs affect object decoding accuracy. To answer this, we recorded local field potentials using a laminar microelectrode in macaque IT. We presented two familiar stimuli each for 500 ms successively with an inter-stimulus interval of 500 ms. Trials consisted either of a repetition of the same stimulus or of their alternation. Machine learning-based classifier was employed to decode stimulus identity from the LFP power in different frequency bands of each penetration. We found that the object classification accuracy depended strongly on spectral frequency, with frequencies below 30 Hz (alpha and beta) producing greater accuracies than gamma bands. However, the effect of repetition on classification accuracy was stronger at the gamma frequencies, showing a decrease in classification accuracy for repeated stimuli and a tendency for an improved object encoding when the stimulus was preceded by a different stimulus. The present results demonstrate that due to adapting input, stimulus encoding in IT (1) can be more accurate for stimuli that differ from recently preceding ones while being impaired for stimuli that are repeated, and (2) these effects are more pronounced at high spectral frequencies of the LFP.

## Introduction

The average response of macaque inferior temporal (IT) neurons decreases with stimulus repetition [1–15]. This “repetition suppression” [16] or “adaptation” effect [17] has aroused recent interest because of the widespread use of adaptation paradigms in human fMRI studies [18,19].

Recently, we examined how the repetition-induced changes in IT spiking activity affect the accuracy by which IT neurons encode objects [20]. We compared the discriminability of stimuli presented in either the first (adapter) or second (test) position in sequences of two serially presented stimuli. We found that the single unit discriminability of repeated familiar stimuli was reduced compared to non-repeated stimuli. However, in some conditions for which adapter and test shapes differed, the cross-adaptation resulted in an enhanced discriminability. This decreased discrimination accuracy for repeated compared to non-repeated stimuli was confirmed when examining the multi-unit activity (MUA) to repeated and non-repeated presentations to two familiar stimuli. Using the spiking activity of the neuronal populations, recorded with a laminar electrode, we showed a decreased classification accuracy for repeated compared to non-repeated test stimuli, but classification was enhanced for the test compared to adapter stimuli when the test stimulus differed from recently seen stimuli. These findings suggested that adaptation in IT supports efficient coding of stimuli that differ from recently seen ones but impairs the coding of repeated stimuli. Note that these effects of repetition on object classification accuracy may hold only for short duration adaptation and/or short delay intervals between adapter and test stimulus, i.e. for short-term adaptation.

Here, we examine the effects of such short-term adaptation on the classification accuracy for a second measure of neural activity, local field potentials (LFPs). LFPs represent a population measure of neuronal, mainly synaptic and dendritic, activity in the local cortical network [21–23]. Measuring adaptation in LFPs and comparing adaptation effects for LFPs and spiking activity is important for at least three reasons. First, since adaptation affects response to a stimulus within an entire network of neurons, and may alter network properties [15,24], it is of interest to examine how adaptation affects the population encoding accuracy as captured by LFPs. Second, studies suggest that LFPs are better correlated with BOLD than is spiking activity [25–28] and thus knowledge of how repetition affects object encoding by LFPs is relevant for understanding the neural correlate of adaptation-induced changes of BOLD-based object classifications using multi-voxel pattern analysis tools [29]. Thus far no study examined the effect of short-term adaptation on LFP-based object classification. Third, LFPs are believed to reflect an input signal to the neurons at least for frequencies <50 Hz [23,30]. Thus, the classification of stimulus identity from LFPs can provide an insight into the adaptation effects on stimulus coding of the neuronal input. This in turn will complement the stimulus coding results observed for the neuronal output as captured by MUA [20].

Previous studies observed repetition suppression for both spiking activity and LFPs, particularly for the spectral frequencies above 60 Hz [13–15]. LFPs represent a population measure of mainly synaptic activity with different underlying processes for low and high frequency bands [31,32]. Hence, we classified the LFP power to the adapter and test stimuli of different frequency bands (ranging from alpha to high gamma bands), allowing a comparison between repetition effects on the classification accuracies computed from the frequency band-limited LFP power.

## Materials and Methods

### Subjects

Two rhesus macaques (*Macaca mulatta*; male monkey G and female monkey K, weighing 7.2 and 7.6 kg, respectively, both left hemisphere) served as subjects. Animal care and experimental procedures met the national and European guidelines and were approved by the Ethical Committee of the KU Leuven Medical School.

Details about implants and surgery can be found in [20]. The localization of the plastic recording chamber was guided and verified by magnetic resonance imaging (MRI) scans. Recording positions were estimated based on the MRI visualization of glass capillaries filled with the MRI opaque copper sulfate (CuSO_4_) inserted into the recording chamber grid at predetermined positions combined with the microdrive depth readings of the white/gray matter transitions relative to the grid base.

Recordings were made from the lower bank of the superior temporal sulcus (STS). The anterior–posterior coordinates of the estimated recording positions ranged between 16 and 18 mm, and 15 and 17 mm anterior to the auditory meatus in monkeys G and K, respectively. The medial-lateral coordinates ranged between 22 and 24 mm, and 20 and 21 mm lateral to the midline in monkeys G and K, respectively. These are the same penetrations which were originally made to study the effect of stimulus repetition on the synchrony of macaque IT cortical activity [15] and in addition were used to address the question how adaptation affects object representation accuracy at the level of MUA in macaque IT cortex [20].

### Recordings

LFPs were recorded using a 16-channel Plextrode U-Probe (Plexon Inc.). The inter-contact (channel) spacing was 100 µm with electrode sites linearly arranged on a single shaft (outer diameter of 185 µm). The U-Probe was lowered with a Narishige microdrive through a guide tube. The grounded guide tube and metal shaft served as the reference. Recordings were made using a Plexon data acquisition system. Recorded signals were preamplified with a headstage having an input impedance of >1GΩ. The signals were split into spiking activity (band-passed signal between 250 Hz and 8 kHz) and LFPs (band-passed signal between 0.7 and 170 Hz obtained by applying to the signal a high-pass two-pole Butterworth filter with a cut-off frequency of 0.7 Hz and a low-pass four-pole Butterworth filter with a cut-off frequency of 170 Hz and followed by digitization at 1 kHz).

The U-Probe was positioned so that visually-driven MUA was present on most if not all channels and LFP response to the presented stimuli was clearly visible for each channel. After positioning the U-Probe in the STS, we waited for approximately 2 hours before performing the recordings to ensure good recording stability.

Eye position was measured online with an infrared-based eye tracking system (ISCAN EC-240A, ISCAN Inc.; 120 Hz sampling rate). The analog eye movement signal was saved using a sampling frequency of 1 kHz. Eye positions, stimulus and behavioral events were stored for later off-line analysis on a computer which was synchronized with the Plexon data acquisition system.

### Stimuli and tests

The stimulus set consisted of 52 color images including human and monkey faces, human and monkey bodies, body parts, mammals, birds, fish, snakes, spiders, trees, fruits, fractals and manmade objects. The maximum size of the objects was approximately 5° of visual angle. The stimuli were presented on a uniform gray background with their centers of mass positioned in the center of a CRT display (frame rate 60 Hz) located 61 cm from the subject’s eyes.

**Figure 1:**
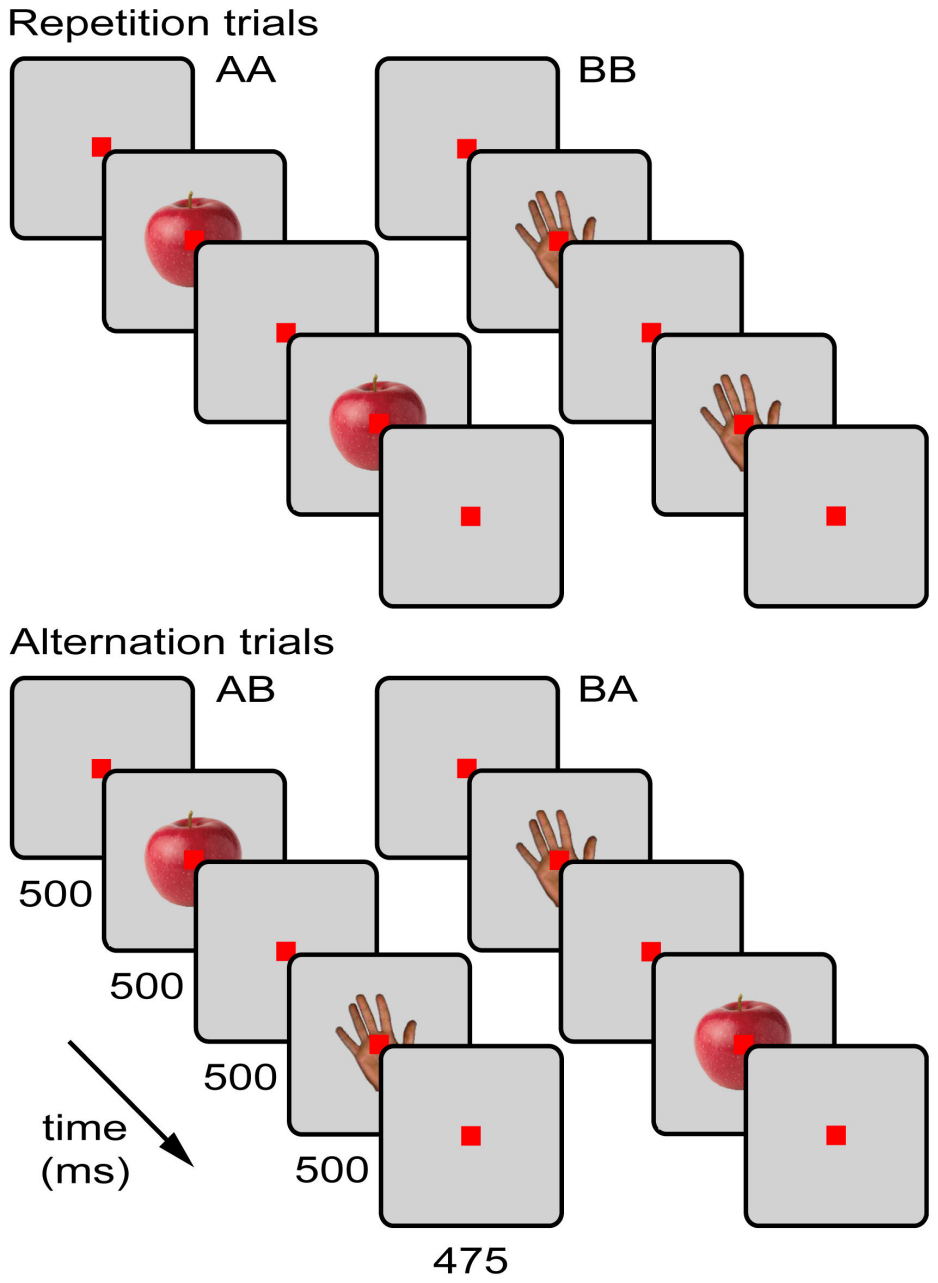
Adaptation test. Trials consisted of the successive presentations of either two identical (repetition trials, AA and BB) or two different (alternation trials, AB and BA) stimuli, each presented for 500 ms and separated by a blank screen for 500 ms. Monkeys initiated a trial by passively fixating for 500 ms a red target square (size: 0.17°, here shown not to scale), which was presented in the center of the monitor and remained visible throughout an entire trial. Continuous fixation on the target square during the stimulus presentations and 475 ms after the test stimulus offset resulted in a fluid reward delivered to the monkeys.

The two images to be used during the adaptation test were selected by means of a preliminary test. We presented the 52 images while the animal was performing the passive fixation task during which the stimuli were shown for a duration of 500 ms. Based on the spiking responses to the stimuli in the different channels, we selected in each penetration two images which elicited a response in most of the 16 channels throughout the thickness of cortex. Next, using these two selected images, A and B, we ran the adaptation test ( [15]; Figure 1) in which two stimuli, adapter and test, were presented for 500 ms each, separated by a blank screen (ISI) for 500 ms. The stimuli within a trial were either the same (AA or BB trials, repetition trials) or different (AB or BA trials, alternation trials) images. Subjects were required to maintain fixation from 500 ms prior to the adapter stimulus onset until 475 ms after the test stimulus offset. Continuous fixation in this 2475 ms interval was followed by a fluid reward. Any break in fixation during this interval aborted the trial.

The fixation window sizes ranged from 1.1 to 1.8° horizontally and 1.6 to 2.6° vertically across the monkeys. Aborted trials were not analyzed further. The time interval between the test stimulus offset and the adapter stimulus onset for the next trial or, in the case of aborts, between the end of the aborted stimulus and the beginning of the adapter stimulus of the next trial varied across trials since it depended on the oculomotor behavior of the animal. The medians of these time intervals ranged from 3326 to 3494 ms across monkeys, with minima ranging from 2856 to 2923 ms. These values are well above the 500-ms ISI of a stimulus sequence. The order of the four different trial sequences (AA, BB, AB and BA) was pseudorandomized with the constraint that the adapter image of a trial always differed from the last presented image of the preceding unaborted or aborted trial. The proportion of AA, BB, AB and BA unaborted trials was similar. The mean number of trials per condition across penetrations was 123.5 (minimum = 86 trials, maximum = 156 trials).

### Data analysis

LFPs were filtered offline with a digital 50-Hz notch filter (48-52 Hz fourth-order Butterworth FIR filter; Fieldtrip Toolbox, F.C. Donders Centre for Cognitive Neuroimaging, Nijmegen, The Netherlands; http://www.ru.nl/fcdonders/fieldtrip). Trials in which the signal was <1% or >99% of the total input range were excluded (median % removed trials across all conditions and animals: 0.4%). By convolving single-trial data using complex Morlet wavelets and taking the square of the convolution between the wavelet and signal [33], the time-varying power of the signal for every frequency was obtained. The complex Morlet wavelets had a constant center frequency-spectral bandwidth ratio (*f*_0_ / *σ*_*f*_) of 7, with *f*_0_ ranging from 1 to 170 Hz in steps of 1 Hz. Only sites for which the spiking activity showed a significant response to either A or B presented as adapter or test stimulus entered further LFP analysis (see 20). Since a previous analysis of the same recordings [15] showed that the adaptation was stronger in the early than in the late phase of the response to a stimulus, we employed an early analysis window that ranged from 60 till 310 ms poststimulus onset. We refer to [15] for an in-depth analysis of the power spectra of the LFPs of the same data.

The spectral power of each trial was averaged within the early analysis window and each of the following frequency bands: 8-12 Hz (labeled “alpha”), 13-30 Hz (“beta”), 31-60 Hz (“low gamma”), 61-100 Hz (“middle gamma”) and 101-170 Hz (“high gamma”).

### Stimulus decoding from the LFPs spectral power

We classified the two stimuli, A and B, using LFPs’ spectral power. The classification analysis was performed for the following conditions: (1) adapter stimuli, separately for repetition and alternation trials (labeled “Adapter”), (2) test stimuli in repetition trials (“Test(AA, BB)”), (3) test stimuli in alternation trials (“Test(AB, BA)”), and (4) test stimuli following the same adapter stimulus (e.g. A following A versus B following A; “Test(AA, AB)”). The classification analysis of the latter condition was performed separately for the AA versus AB and BB versus BA trial combinations. Since the classification scores did not differ for the adapter stimulus in repetition and alternation trials as well as for the test stimuli in AA versus AB and BB versus BA trial combinations in either monkey (two-sided Wilcoxon matched pairs test, Bonferroni-corrected for two monkeys per frequency band *p* < 0.025), we averaged those per condition. Because of the constraint that the adapter stimulus needed to differ from a lastly presented stimulus of the preceding trial, the AB and BA sequences were likely to occur further apart in time compared to the AA and BB sequences. Note that the absence of a difference in the classification scores for the adapter stimulus in repetition and alternation trials also implies no effect of the difference in the conditional probabilities of these two types of sequences.

For each condition and penetration, we made a neuronal population consisting of the simultaneously recorded sites of that penetration (median number of sites per penetration = 15.5; range: 11-16). In order to compare classification accuracies across the four conditions, we equated the number of trials per stimulus for these conditions that were used to train and test the classifiers. Thus, for each penetration, the number of trials per stimulus (N_tot_) that entered the classifier was equal to the smallest even number of trials per stimulus of the four conditions of that penetration. The mean N_tot_ was 112 trials and ranged from 84 to 122. Half of the N_tot_ trials were used for training, while the remaining half of the trials was used for testing the classifier. All the reported classification accuracies are based on the classifications obtained during testing (cross-validated classification scores). For each condition and penetration, we trained and tested 1000 classifiers by randomly drawing for each classifier the training and test trials from the pool of all available trials. The classification scores of a population of sites of a penetration are the averages of these 1000 classification scores.

We employed Support Vector Machines (SVM; [34,35]) which perform classifications by constructing a hyperplane in a multidimensional space that separates items, here single-trial LFP power of a particular frequency band, of different class labels, here A and B stimuli. We used a linear SVM since it is a relatively simple classifier and less susceptible to overfitting than non-linear SVM (e.g. [36]). The SVMs were performed using the Matlab “svmtrain” function with default parameters (quadratic programming method was used in order to find the separating hyperplane) of the Matlab Bioinformatics toolbox. The training and test data for each site were standardized by subtracting the mean (averaged across both stimuli for the training trials) from each response and dividing this difference by the standard deviation of the responses of the training data (z-normalization).

As a control, we ran the classifiers on the label-shuffled data in which the stimulus labels (A or B) were randomly permuted across the trials. This permutation of the stimulus labels was performed 1000 times and 1000 classifiers were trained. As expected, the mean percent correct classification performance for the shuffled data was 50%.

To compare the results of classification of stimulus identity from LFPs for different frequency bands to those when using MUA [20], we ran the SVM classification analysis for MUA of the same penetrations. This classification analysis for MUA was identical to that used for the decoding of stimulus identity from the LFPs spectral power. Instead of using the averaged power in a particular frequency band as the response to a stimulus, the classification analysis for MUA was based on raw spike counts in the early analysis window.

Power in spectral frequencies above 50 Hz can be contaminated by low frequency residuals of simultaneously recorded spikes. As in the large majority of other LFP studies (e.g. [37–41]), we did not attempt to remove those spikes residuals for several reasons. First, we feel that in order to link LFPs to the BOLD signal, one should consider the full LFP signal and not one in which spikes residuals are removed. The second and most important reason is that by removing spikes from the LFPs we would have considerably deteriorated the signal, since we recorded multi-unit activity [20]. Indeed, applying any known method to remove spike residuals from the LFP signal would be rather detrimental that beneficial for our data set. Each such method operates by extrapolating the LFP signal in a small window around detected spikes. The span of this window varies from 1.5 to 3 ms (e.g. [42,43]). Given the high firing rates in our data (peak values were approximately 75 and 85 spikes/sec in monkeys G and K, respectively; for details, see 20) we believe that such extensive interpolation of LFPs will considerably deteriorate the signal. Third, spike removal procedures are essential when computing spike-triggered LFP averages, phase locking of spikes and spike-LFP coherence where spikes and LFPs are measured with the same electrode [43]. We did not apply any of these analyses.

### Analysis of eye movements

The results of analyses of eye movements, including microsaccade rates, are reported in [15]. They demonstrated that the stimulus-selective repetition suppression of the neural responses cannot be explained by eye movement differences between adapter and test stimuli.

## Results

We recorded LFPs using a laminar electrode located in IT during an adaptation paradigm in which repetitions of the same images (AA or BB sequences) were randomly interleaved with successive presentations of different images (AB or BA sequences). We made 21 and 11 penetrations in monkeys G and K, respectively, yielding 319 and 149 sites with responsive MUA to either stimulus A or B in monkeys G and K, respectively. The LFPs from these responsive sites were analyzed further (only MUA-responsive sites were employed for the analyses, ensuring that the recordings were performed in the gray matter).

**Figure 2:**
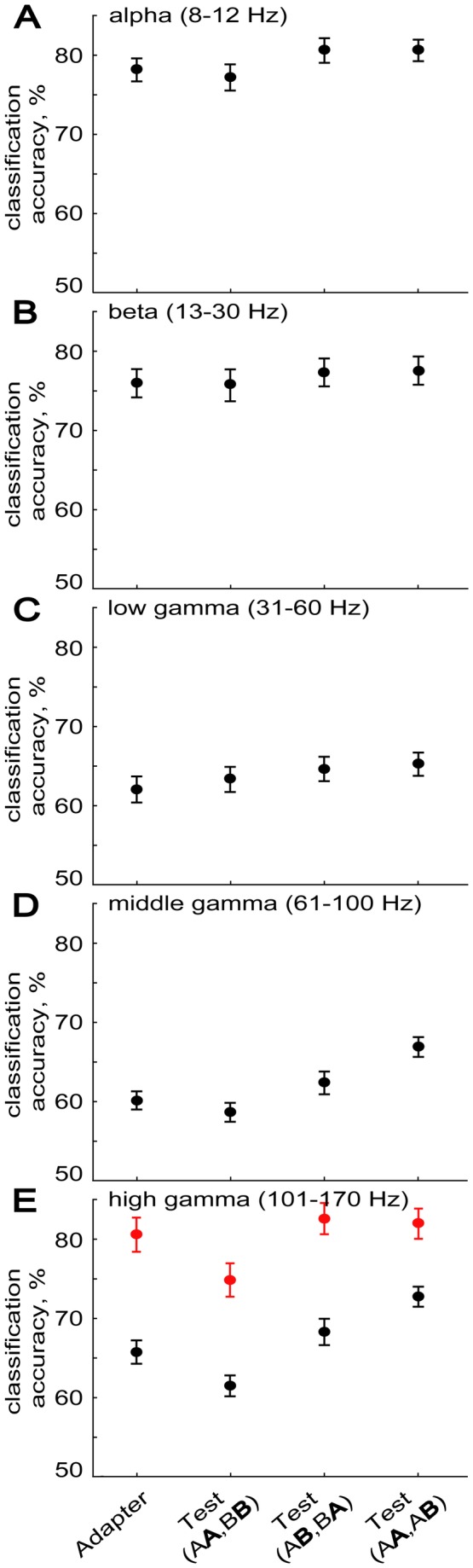
Classification accuracies for the multi-unit activity (in red) and frequency band-limited LFP power (in black). Mean classification accuracies (N = 32 penetrations) for the four stimulus conditions listed on the abscissa are plotted for five frequency bands: (A) alpha (8-12 Hz), (B) beta (13-30 Hz), (C) low gamma (31-60 Hz), (D) middle gamma (61-100 Hz) and (E) high gamma (101-170 Hz). Support Vector Machines were trained and tested for each condition and frequency band separately. Error bars indicate standard error of the mean.

After Morlet wavelet transform, we averaged the spectral power in each of the five frequency bands and in the response window. SVM classifiers were trained and tested for each band and stimulus condition separately. Figure 2 shows the mean classification scores for each frequency band as a function of stimulus condition. Classifiers were trained separately for each simultaneously recorded population of sites per penetration. Thus, the mean classification scores refer to the classification scores, averaged across the penetrations. For each frequency band, the classification scores were well above the chance level (50%). However, the classification scores strongly depended on the frequency band (repeated measures ANOVA with frequency band and stimulus condition as factors; *F*(4,120) = 51.156, *p* < 0.0001) with a significant interaction of frequency band and stimulus condition (*F*(12,360) = 7.0320, *p* < 0.00001). The highest classification scores were obtained for the alpha band, followed closely by the beta band (Figure 2). The high accuracy for these low frequency bands is not that surprising since the LFP waveforms differed between stimuli (see 15) and these differences in waveforms are reflected in the power at these low frequencies. For comparison with the classification scores obtained for the LPFs spectral power, Figure 2 also shows the mean classification scores for the MUA (in red) recorded from the same sites of the same penetrations.

The alpha, beta and low gamma band classifications showed a significant effect of stimulus condition (one-way repeated measures ANOVA; alpha: *F*(3,93) = 9.2488, *p* < 0.00002; beta: *F*(3,93) = 3.2921, *p* < 0.05; low gamma: *F*(3,93) = 6.8069, *p* < 0.0005) with the mean classification scores for the test stimulus in the alternation trials (Test(AB, BA)) and in the Test(AA, AB) condition both larger than those for the adapter and the test stimulus in repetition trials (Test(AA, BB)). However, these effects were numerically small (about 2-3% difference; Figure 2) and survived Bonferroni Post Hoc testing only for the alpha band (*p* < 0.05). For the low gamma, Bonferroni Post Hoc testing showed significant effects only for the Adapter condition versus Test(AB, BA) and Test(AA, AB) (*p* < 0.01).

**Figure 3:**
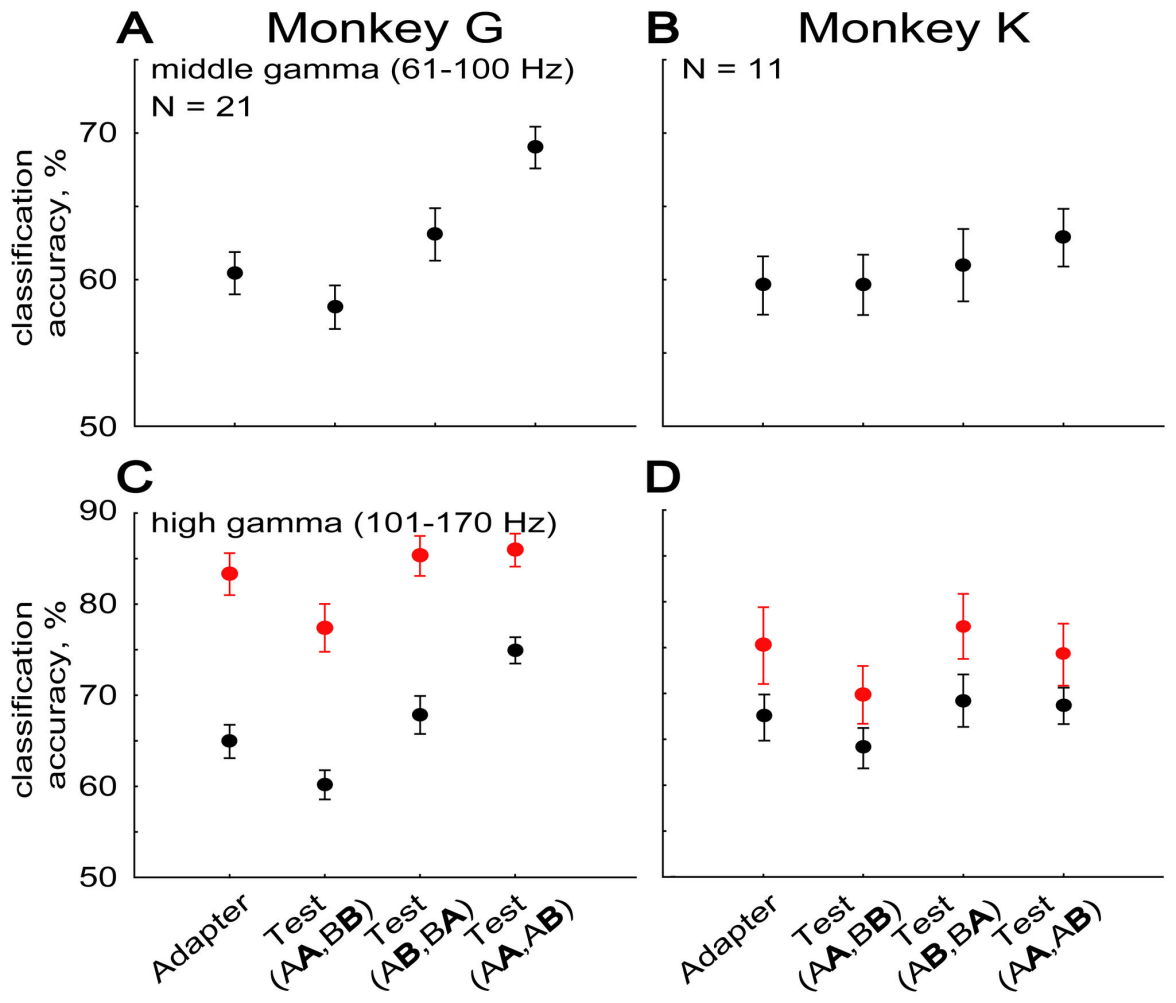
Classification accuracies for the multi-unit activity (in red) and the middle and high gamma LFP power (in black) in each of the two monkeys. (A,C) Monkey G, (B,D) monkey K.N indicates the number of penetrations, the sites of which (up to 16 per penetration) were employed for classifications. Error bars indicate standard error of the mean.

The middle gamma band classification accuracies also showed a significant effect of stimulus condition (one-way repeated measures ANOVA; *F*(3,93) = 32.609, *p* < 0.00001). As for the lower frequency bands, the classification scores for the test stimulus in the alternation trials (Test(AB, BA)) and in the Test(AA, AB) condition were larger than those for the test stimulus in repetition trials (Test(AA, BB); Bonferroni Post Hoc test, each *p* < 0.0005). However, the most remarkable effect was the significantly higher accuracy score for the test stimulus in the Test(AA, AB) condition compared to the other three conditions (each *p* < 0.00005), including the test stimulus in alternation trials (Test(AB, BA)). This higher accuracy for the Test(AA, AB) condition was present in each animal (Figure 3A,B).

The high gamma band classification showed numerically the strongest effect (11% difference) of stimulus condition (one-way repeated measures ANOVA; *F*(3,93) = 34.967, *p* < 0.00001). Similar to the effects seen for the middle gamma band, the highest mean classification accuracy for the high gamma power was observed for the test stimuli in the Test(AA, AB) condition, which differed significantly from the three other conditions (Bonferroni Post Hoc test, each *p* < 0.001). Contrary to the lower frequency bands but similar to what we observed when classifying the spiking activity at the same sites [20], the classification accuracy was significantly lower for the test stimulus in repetition trials (Test(AA, BB)) compared to the adapter (*p* < 0.005) and the test stimuli in the two other conditions (Test(AB, BA) and Test(AA, AB); *p* < 0.000001). Thus, stimulus repetition reduced the classification accuracy in this high frequency band. The classification accuracy for the adapter and test stimuli in alternation trials (Test(AB, BA)) did not differ significantly. Thus, the effects for the high gamma band were similar to those observed for the spiking activity, except for the relatively high classification scores for the test stimuli in the Test(AA, AB) condition. However, unlike the other effects, this increase for the Test(AA, AB) condition was present in monkey G but not in the other animal (Figure 3C,D; repeated measures ANOVA with monkey and stimulus condition as factors; interaction monkey and stimulus condition: *F*(3,90) = 9.2102, *p* < 0.00005).

In our analyses of classification accuracy, we decoded stimulus identity based on the responses to a presented stimulus (A or B) at each analyzed electrode site per penetration. Given that the maximum distance between the sites was 1.5 mm while similar LFPs selectivity has been observed in a larger area (e.g. [44]), it is possible that information on stimulus identity distributed across all analyzed sites per penetration is redundant due to this shared LFPs selectivity across sites. If the responses to a stimulus were correlated across sites, one would expect to achieve similar classification accuracy when reducing the number of analyzed sites per penetration. On the contrary, if reducing the number of sites resulted in a decrease of classification accuracy, this would indicate that despite the shared LFPs selectivity each site provided additional information on stimulus identity. To test this hypothesis, we classified the identity of the adapter stimulus in repetition trials employing only 1/3 of the electrode sites per penetration that entered the original analysis (median number of sites per penetration = 5.0; range: 3-5). For all frequency bands, we observed a significant decrease in classification accuracy when reducing the number of analyzed sites (mean classification accuracy across both monkeys for the whole and reduced sets of analyzed sites per penetration, respectively: alpha: 78.1% versus 71.2%, *p* < 0.000005, two-sided Wilcoxon matched pairs test; beta: 75.9% versus 72.4%, *p* < 0.000005; low gamma: 62.4% versus 60.6%, *p* < 0.005; middle gamma: 60.2% versus 58.1%, *p* < 0.0005; high gamma: 66.0% versus 62.7%, *p* < 0.00005). These findings show that the LFP signals per site were at least to some extent independent.

## Discussion

Classification accuracy for the alpha and beta bands was comparable to that of the MUA measured at the same sites ( [20]; compare black bars in Figure 2A,B with red bars in Figure 2E) and higher than that obtained for the higher frequency bands. This greater classification accuracy for low compared to high frequencies has also been observed in anesthetized macaque V1 using natural movies as stimuli [32]. It may at least partially be due to differences in luminance and contrast and hence overall stimulus drive. Indeed, in our and the V1 study [32], stimuli differed in luminance. It still remains to be examined whether such high classification accuracies for the low frequency bands are also present when employing stimuli equated for these low-level image properties.

To our knowledge, we present here the first analysis of stimulus classification of LFP power in an adaptation paradigm. For the alpha, beta, low and middle gamma bands, classification accuracy was statistically indistinguishable for the adapter and test stimuli in repetition trials. This novel finding suggests that classification accuracy based on low spectral frequency LFP signals is not reduced when repeating a stimulus, which contrasts with the marked decrease in classification accuracy of MUA ( [20]; red bars in Figure 2E) for the test stimulus in the same repetition trials. However, a significant decrease in classification accuracy for repeated stimuli was present for the high gamma band in both animals, which agrees with previous observations of a correlation between high gamma power and MUA ( [45–47]; compare black and red bars in Figure 3C,D).

We wish to stress that the adaptation paradigm we employed assesses short-term repetition or adaptation effects. Such short-term adaptation effects may well differ from those seen after long duration adaptation (e.g. [29,48,49]) or after long delays. Furthermore, the stimuli employed in the present study were all familiar to the monkey since they were employed to search for responsive units. Interestingly, in a study from Sheinberg’s group [50] classification accuracy based on LFPs recorded in macaque IT was found to be higher for familiar compared to novel stimuli. This contrasts with our finding of lower classification accuracy in the middle and high gamma bands for repeated versus non-repeated stimuli, indicating that the short-term adaptation effects differ from long-term, learning-related familiarity effects.

The higher classification accuracy for the Test(AA, AB) condition compared to the other conditions for higher frequencies is noteworthy and unexpected. It was present in both animals for the middle gamma frequencies but present only in one animal for the high gamma frequencies. The classification accuracy in the Test(AA, AB) is the average of classification scores for the test stimuli across AA versus AB and BB versus BA trial combinations (see Materials and Methods). For the sake of argument, assume that stimulus A evokes a higher response than stimulus B. Repetitions of stimulus A (AA trial sequence) will result in a decreased response to the repeated A while largely preserving the response to stimulus B following A. This will decrease the difference in response between those stimuli and, when assuming this difference to be a major determinant for the decoding of stimulus identity, result in a poorer classification accuracy compared to that for the adapter. On the other hand, the reduced response to the repeated B (BB trial sequence) and the largely unchanged response to A when preceded by B will lead to a greater difference in response between these stimuli and result in a better classification accuracy compared to that for the adapter. Thus, the increased classification accuracy in the Test(AA, AB) condition is not trivial.

Although this increase was only present in one animal for the high gamma frequencies (Figure 3), it does suggest that the power in those high frequency bands, even above 100 Hz, does not merely reflect MUA. Thus, classification of MUA in adaptation paradigms does not always produce the same effects as those seen in high frequency LFP power, although in many studies both measures were well correlated. A discrepancy between spiking activity and high gamma tuning has also been observed in MT and MST [51]. One possibility is that the high gamma power – as does the low frequency power –also reflects synaptic activity and that this input shows a high sensitivity for a repeated versus a non-repeated test stimulus. Whatever the reasons for the discrepancy between MUA and high gamma power are, our data demonstrate that it is informative to examine high frequency LFP power in addition to MUA.

Because the fMRI BOLD response correlates with LFPs [25–28], the present data are relevant for a recent human fMRI study that examined the effect of stimulus repetition in ventral stream areas using multi-voxel pattern classification [29]. These authors reported a (non-significant) tendency for lower category classification accuracy for blocks of repeated compared to unrepeated stimuli in ventral temporal cortex, which might be a homologue of macaque IT. The same trend was present in the alpha, middle and high gamma bands in the present macaque IT study, but most pronounced and significant for the high spectral frequencies. This correspondence between BOLD and high gamma band power classification accuracy is in line with previous studies that observed a positive correlation between gamma band power and BOLD signal in primates [52–54]. Note that BOLD does not always reflect gamma band power since [27] showed that during perceptual suppression, BOLD responses were related to low instead of high frequency LFP power. Indeed, the coupling of the hemodynamic signal to neural activity can be task- and context-dependent [55,56].

Higher on average accuracy for the test stimuli in alternation trials compared to that for the adapter stimuli tended to be present (but not always significant) in all frequency bands. It should be noted that even in the alternation trials, the power for the adapter and test stimuli differed [15] due to so-called cross-adaptation [13]. Such cross-adaptation indicates that at least part of the neuronal population driving LFPs receives input from both stimuli, either bottom-up or indirectly through lateral connections or feedback from higher regions. The present results show that due to such adapting input, stimulus encoding in IT can be more accurate for stimuli that differ from recently preceding ones.
