# Correction: Decoding of Repeated Objects from Local Field Potentials in Macaque Inferior Temporal Cortex

**DOI:** 10.1371/annotation/411033ae-4884-42db-826f-b9dda6564860

**Published:** 2013-09-10

**Authors:** Dzmitry A. Kaliukhovich, Rufin Vogels

There was an error in the name of the first author. The correct name is: Dzmitry A. Kaliukhovich 

